# A Potential New Pathway for *Staphylococcus aureus* Dissemination: The Silent Survival of *S. aureus* Phagocytosed by Human Monocyte-Derived Macrophages

**DOI:** 10.1371/journal.pone.0001409

**Published:** 2008-01-09

**Authors:** Malgorzata Kubica, Krzysztof Guzik, Joanna Koziel, Miroslaw Zarebski, Walter Richter, Barbara Gajkowska, Anna Golda, Agnieszka Maciag-Gudowska, Klaudia Brix, Les Shaw, Timothy Foster, Jan Potempa

**Affiliations:** 1 Department of Microbiology, Faculty of Biochemistry, Biophysics and Biotechnology, Jagiellonian University, Krakow, Poland; 2 Department of Immunology, Faculty of Biochemistry, Biophysics and Biotechnology, Jagiellonian University, Krakow, Poland; 3 Department of Cell Biophysics, Faculty of Biochemistry, Biophysics and Biotechnology, Jagiellonian University, Krakow, Poland; 4 Center for Electron Microscopy of the Medical Faculty, Friedrich-Schiller-University Jena, Jena, Germany; 5 Department of Cell Ultrastructure, Medical Research Centre, Polish Academy of Sciences, Warsaw, Poland; 6 School of Engineering and Science, Jacobs University Bremen, Bremen, Germany; 7 Department of Biology, University of South Florida, Tampa, Florida, United States of America; 8 Department of Microbiology, Moyne Institute of Preventive Medicine, Trinity College, Dublin, Ireland; University of Birmingham, United Kingdom

## Abstract

Although considered to be an extracellular pathogen, *Staphylococcus aureus* is able to invade a variety of mammalian, non-professional phagocytes and can also survive engulfment by professional phagocytes such as neutrophils and monocytes. In both of these cell types *S. aureus* promptly escapes from the endosomes/phagosomes and proliferates within the cytoplasm, which quickly leads to host cell death. In this report we show that *S. aureus* interacted with human monocyte-derived macrophages in a very different way to those of other mammalian cells. Upon phagocytosis by macrophages, *S. aureus* persisted intracellularly in vacuoles for 3–4 days before escaping into the cytoplasm and causing host cell lysis. Until the point of host cell lysis the infected macrophages showed no signs of apoptosis or necrosis and were functional. They were able to eliminate intracellular staphylococci if prestimulated with interferon-γ at concentrations equivalent to human therapeutic doses. *S. aureus* survival was dependent on the alternative sigma factor B as well as the global regulator *agr*, but not SarA. Furthermore, isogenic mutants deficient in α-toxin, the metalloprotease aureolysin, protein A, and sortase A were efficiently killed by macrophages upon phagocytosis, although with different kinetics. In particular α-toxin was a key effector molecule that was essential for *S. aureus* intracellular survival in macrophages. Together, our data indicate that the ability of *S. aureus* to survive phagocytosis by macrophages is determined by multiple virulence factors in a way that differs considerably from its interactions with other cell types. *S. aureus* persists inside macrophages for several days without affecting the viability of these mobile cells which may serve as vehicles for the dissemination of infection.

## Introduction

*S. aureus* is a leading etiologic agent of nosocomial and community-acquired infectious diseases. The pathogenesis of staphylococcal disease usually proceeds from a local infection (e.g., wound infection, furuncle and cellulitis) to systemic dissemination (bacteremia) and then to metastatic infections (e.g., endocarditis, osteomyelitis and septic arthritis). Toxinoses (e.g., toxin shock syndrome, scaled skin syndrome, and food-borne gastroenteritis) constitute a separate group of staphylococcal diseases resulting from the local or systemic effects of specific toxins [1], [2].

The public health concern regarding staphylococcal infections is magnified by the increasing prevalence of multiply antibiotic resistant strains such as methicillin-resistant *S. aureus* (MRSA) and glycopeptide-insensitive *S. aureus* (GISA) [3]. The appearance of vancomycin-resistant strains (VRSA) suggests the possibility of a return to a pre-antibiotic era where 80% of bloodstream infections were fatal. A comprehensive understanding of the pathogenic pathways exerted by *S. aureus* is urgently needed to aid the design of new therapeutic and preventive treatments.

The success of *S. aureus* as a pathogen is primarily due to its ability to produce a large number of virulence factors. These include several MSCRAMMs (**m**icrobial **s**urface **c**omponents **r**ecognizing **a**dhesive **m**atrix **m**olecules) that bind fibrinogen, fibronectin, laminin, collagen, vitronectin, and thrombospondin to promote colonization [4]. Host defenses are thwarted by the production of microcapsule, protein A, coagulase, complement inhibitors, fatty acid-metabolizing enzymes and leukocidin while proteases, nucleases, lipases, hyaluronate lyase and staphylokinase facilitate tissue invasion [5]. Secreted toxins (e.g. toxin shock syndrome toxin, enterotoxins and exfoliative toxins) elicit sepsis and/or induce specific toxinoses. Nevertheless, despite the recognition of this plethora of virulence factors, we are still far from identifying exactly which ones are necessary for infection and which ones may be targeted by new therapies [6].

*S. aureus* can modulate the transcriptional activity of virulence genes in response to environmental changes controlled by global regulatory elements that are of two major types; two-component regulatory systems and the SarA family of DNA binding-proteins [7]. A major two-component regulatory system is encoded by the *agr* locus which controls the expression of many virulence factors including proteases, MSCRAMMs and toxins in a growth phase and density-dependent manner [8]. The synthesis of many cell-wall associated proteins is stimulated during the initial stages of growth but is repressed post-exponentially. In contrast, the expression of extracellular proteins, including toxins and proteases, is activated during the post-exponential growth phase converting bacteria from adherent to invasive.

Several other regulatory loci control the expression of virulence factors by modulating *agr* expression, including the alternative Sigma factor B (σ^B^) [9], [10]. Such a multi-leveled regulatory system enables *S. aureus* to express virulence factors under varying conditions, which is of clinical significance since mutants of regulatory loci have severely impaired virulence [11]–[13].

Although *S. aureus* is considered to be an extracellular, pyogenic pathogen, an emerging body of evidence indicates that intracellular reservoirs of *S. aureus* may contribute to persistence such as occurs in recurrent staphylococcal rhinosinusitis and to relapses of infection after antimicrobial therapy [2], [14]. Recent *in vitro* studies have revealed that *S. aureus* can invade a variety of non-professional phagocytic cells, including keratinocytes, fibroblasts, endothelial and epithelial cells, enterocytes and osteoblasts [15]–[21]. Bacterial uptake is promoted by fibronectin which acts as a bridging molecule between integrin α5β1 on the cell surface and the *S. aureus* fibronectin-binding proteins [16], [19], [22], [23]. This activates the Src family of protein-tyrosine kinases and stimulates internalization of adherent bacterium by the ‘zipper mechanism’ [24], [25], [26]. Internalized bacteria reside in endosomal vacuoles or are diverted from the endosomal pathway to autophagosomes depending on the cell type invaded and/or the *S. aureus* strain [27]. Subsequently, *S. aureus* escapes into the cytoplasm where it eventually kills the host cell through the induction of apoptosis [15], [18], [20], [28], [29]. Although secretion of α-toxin by internalized bacteria has been shown to kill host cells [18], [30] it is becoming increasingly apparent that other signals from internalized, metabolically active staphylococci are required to induce apoptosis and that this process requires multiple virulence factors [29], [31]–[33].

Professional phagocytes such as neutrophils, macrophages and dendritic cells are designed to actively engulf microbes and kill them. Only a few types of microbial pathogen can survive phagocytosis by neutrophils and macrophages and they do so by using a several distinct mechanisms to avoid destruction in phagolysosomes [34], [35]. Surprisingly, *S. aureus* also appears to be resistant to bactericidal attack inside the phagocytic vacuoles of neutrophils which can contain viable intracellular bacteria when isolated from sites of infection [36]–[38]. Recent *in vitro* studies confirmed the high level of resistance of *S. aureus* to killing by neutrophils [39]. Furthermore neutrophils can create an environment *in vivo* that promotes intracellular growth of *S. aureus* and contributes to dissemination during infection [40]. Neutrophils, however, are unlikely cells to carry bacteria from the focus of infection into the circulation since they are short-lived [41]. Macrophages are far better suited to this purpose because they are long lived and mobile cells that take phagocytosed material to the lymphoid tissue [42], [43]. If carrying live *S. aureus* macrophages may contribute to the ability of the organism to invade the vascular system from localized infection sites [44]. However, very little is known about the interaction of *S. aureus* with macrophages. *S. aureus* is able to survive in mice and rat macrophages [45]–[48]. In addition, Elliott *et al.* [49] have demonstrated short-term survival of *S. aureus* inside human alveolar macrophages. However, none of these investigations have addressed specifically which bacterial factors are important for intracellular persistence.

The objective of this study was to investigate the long-term ability of *S. aureus* to survive uptake by human monocyte-derived macrophages (hMDMs). We found that significant numbers of *S. aureus* cells which had avoided killing during the first 4–5 days post-phagocytosis were suddenly able to lyse the macrophages in which no signs of apoptosis or necrosis were previously demonstrated. Furthermore these newly escaped *S. aureus* cells were then able to proliferate abundantly in the conditioned media. Bacterial survival and escape from phagocytes was dependent not only on α-hemolysin, but also on functional *agr* and σ^B^ loci, as well as on the expression of sortase A and the metalloprotease, aureolysin. Thus *S. aureus* survived inside macrophages in a metabolically active form until the intracellular environment become suitable for escape. Our findings suggest that the ability of *S. aureus* to survive phagocytosis by human macrophages may contribute to dissemination of the infection and may be detrimental to the host.

## Results

### Infection of macrophages by *S. aureus* does not damage adherent phagocytes

**Figure 1:**
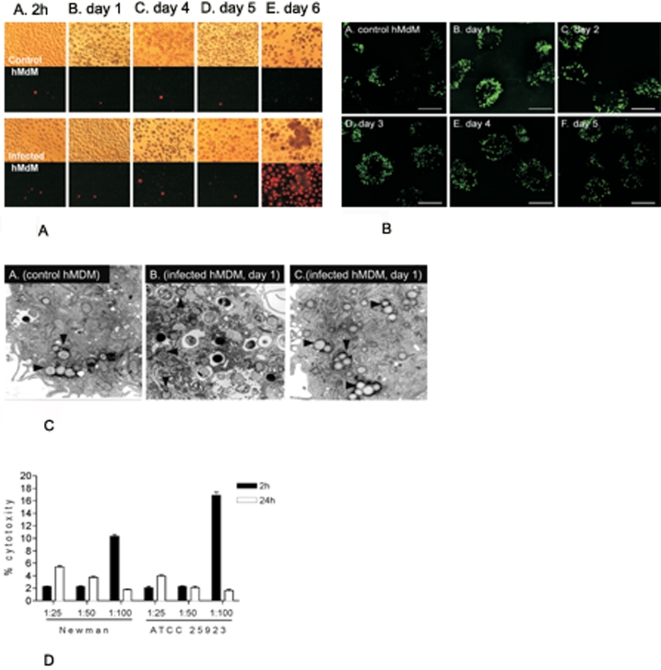
Internalization of *S. aureus* strain Newman does not affect hMDM viability until the plasma membrane is permeabilized. A. Transmission light (upper two rows) and fluorescence (lower two rows) micrographs (x 40) of control (upper panel) and *S. aureus*-infected cells (bottom panel) maintained in culture for 2h (A) and 1 day (B), 4- (C), 5- (D), and 6-days (E). Propidium iodide-positive cells represent infected host cells with leaky plasma membranes. B. BODIPY495/503 staining of lipid droplets of control and *S. aureus* infected hMDM cultures on five consecutive days post-phagocytosis. Cells were permeabilized with 0.2 % Triton X-100 and stained as described in the Materials and Methods section. All scale bars = 10 µm. C. Transmission electron micrographs of control (panel A) and infected cells (panels B and C) one day post-phagocytosis. Black arrowheads point to lipid droplets. Magnification: x10,000 (A and C) and ×7,500 (B). The photographs presented are representative of a minimum of 20 fields observed. D. Cytotoxicity (%) of *S. aureus* infection. Plasma membrane permeabilization or cell lysis induced in macrophage cultures by *S. aureus* infection at different MOI was determined as LDH activity levels. Cytotoxicity was calculated according to the formula: % cytotoxicity = [(experimental value–low control)/(high control–low control)] × 100, where a low control is the LDH activity in the conditioned medium of the control non-infected culture, while the LDH activity in the whole cell culture with cells lysed with detergent (2% Triton X-100) constitutes a high control. An experimental value was the activity in the conditioned medium from the culture infected with *S. aureus*. According to this calculation the control non-infected culture was assumed to show 0% cytotoxicity. All assays were performed in triplicate.

Previous reports have shown that hMDMs and murine macrophages that have ingested virulent bacteria such as *Streptococcus pneumoniae, Shigella flexnerii, Burkholderia pseudomallei, Yersinia ssp., Salmonella typhimurium,* die *by* cytolytic processes that involve cell swelling, plasma membrane disintegration and karyolysis or by apoptosis [50]–[55]. Contrary to this we have found that the phagocytosis of opsonised *S. aureus* strain Newman by hMDMs in ratios bacteria to macrophages of up to 50:1 (multiplicity of infection, MOI = 50), led to a prolonged, survival of bacteria in phagocytes (see below) that did not affect the viability of the host cells. Indeed hMDMs maintained an intact plasma membrane up to 5 days post-phagocytosis as discerned by a lack of staining with propidium iodide (Fig. 1A), an impermeable fluorophore which can only enter cells with compromised plasma membranes. The infected cells were metabolically active for up to 5 days after uptake of *S. aureus* as indicated by a MTT cell proliferation and cytotoxicity assay which measures the reduction of tetrazolium (MTT) into an insoluble formazan product in viable cells (data not shown). Furthermore, the intracellular staphylococci apparently stimulated lipid metabolism since infected cells were stained with Bodipy 493/503, a marker of neutral lipids and lipid droplets, more vividly than control cells. This difference was visible at 1 day post-uptake (Fig. 1B). At this time point the enhanced accumulation of lipid inside infected cells was also apparent by TEM analysis (Fig. 1C).

The integrity of the plasma membrane of hMDMs infected with different MOIs of *S. aureus* was shown by measuring lactate dehydrogenase (LDH) released into the culture media at 2 h and 24 h post-phagocytosis. As shown in Fig. 1D cytotoxicity (%) calculated based on the LDH activity of culture media of hMDMs infected with strains Newman and ATCC 25923 at MOIs of 25 or 50 was only slightly above that of the control. At a MOI of 100 both *S. aureus* strains caused a significant increase in LDH level at 2 h which returned to basal level at 24 h. Apparently, rapid necrotic lysis of a relatively small subset of macrophages occurred, with the majority of cells surviving uptake, as confirmed by microscopic inspection of the hMDM cultures (data not shown). Indeed, the quantitative counts of total cell numbers indicated the significant loss of cells (5–10%) only after the first day postphagocytosis, at MOI = 25 and 50. The loss of cells was higher at MOI = 100, correlating well with the recorded cytotoxicity (Fig. 1D). Based on these results all further experiments were performed at a MOI of 25.

### The bactericidal functions of infected macrophages are preserved

**Figure 2:**
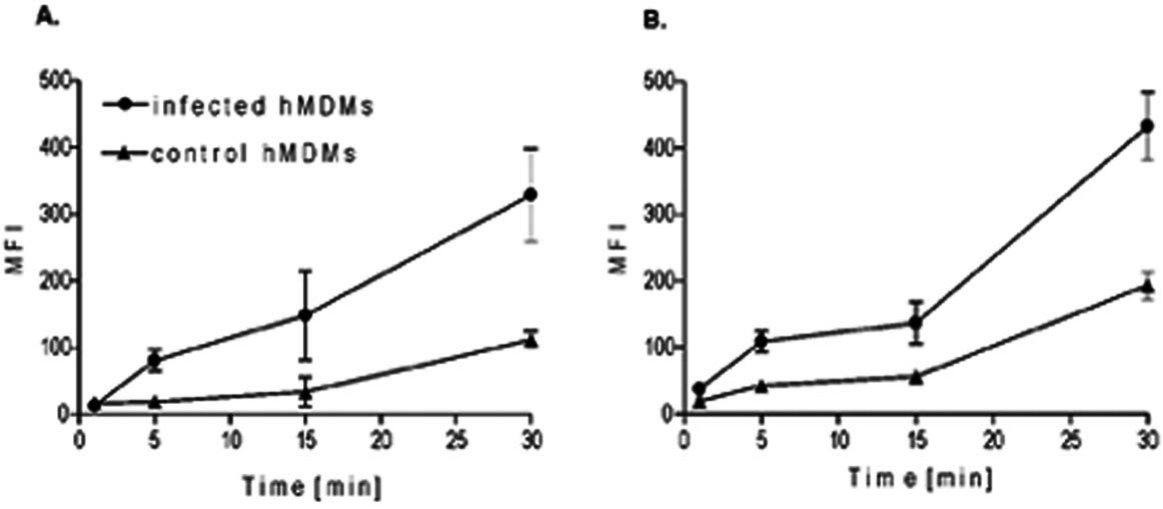
*S. aureus* infected macrophages retained their bactericidal functions. Control hMDMs, and cells 4 days post infection with *S. aureus* strain Newman, were stimulated to phagocytose either live bacteria at a MOI of 1∶25 (1.25×10^7^ CFU) (panel A) or latex beads (panel B). Generation of reactive oxygen species (ROS) determined as the level of the mean fluorescence intensity (MFI) was measured at various time intervals. The data shown is representative of at least three separate experiments, performed in triplicate, using hMDMs derived from different donors.

That the metabolic activity of the infected macrophages was maintained suggested that the bactericidal functions of these cells was also undisturbed. In order to verify this assertion we compared the respiratory burst response of control and infected hMDMs after the phagocytosis of latex beads and live bacteria. As shown in Fig. 2 the kinetics of increased mean fluorescence intensity (MFI), which is a measure of the production of reactive oxygen species (ROS) by cells, was very similar during phagocytosis of both live bacteria (Fig. 2A) and latex beads (Fig. 2B). However, at each time interval the infected hMDMs produced at least twice as much ROS as control cells that took up latex beads or live bacterial cells. Thus, it is clear that infection with *S. aureus* not only does not suppress antibacterial activity of macrophages, but on the contrary, infected cells were primed for a very vigorous response to phagocytic stimuli.

### *S. aureus* persists inside macrophages until the cells lyse

**Figure 3:**
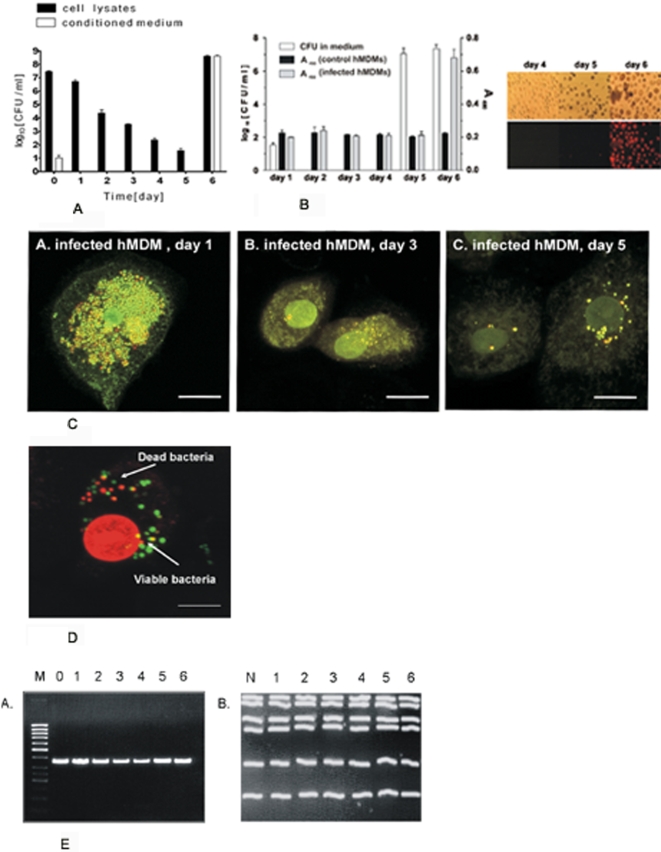
After being phagocytosed by hMDM *S. aureus* strain Newman decreasingly persists intracellularly for several days until a burst of growth on day 6. A. CFU of *S. aureus* in cell lysates and culture medium on six consecutive days post-infection. Macrophages were allowed to engulf *S. aureus* at a MOI of 25 for 2 h, washed, and extracellular bacteria killed by gentamycin. Macrophages were cultured in media without antibiotics. At consecutive days post-phagocytosis media was aspired and hMDMs were lysed. Both conditioned media and cell lysates were plated onto TSA for CFU enumeration. The data shown details of one representative experiment (means±SD) of the 76 we performed in triplicate. B. CFU of *S. aureus* and LDH activity levels (A_490 nm_) in the conditioned culture medium on six consecutive days post infection (left panel); and transmission (upper row) and fluorescent (propidium iodide staining, lower row) light micrographs (x40) of *S. aureus*-infected cells maintained for the indicated time interval post infection. C. Confocal fluorescence microscopy images of hMDMs after 1, 3, and 5 days post-infection at MOI of 25. Cells were fixed, treated with RNase and stained with acridine orange (see Experimental Procedures section). Internalized bacteria are observed as green spots. All scale bars = 10 µm. D. Confocal fluorescence microscopy images of viable bacteria in hMDMs on the 5^th^ day post-phagocytosis. Infected hMDMs were collected, permeabilized with 0.2 % Triton X-100 and double- labeled with propidium iodide and Syto9 (LIVE/DEAD BacLight Kit). Viable *S. aureus* cells are stained green while red signals represent dead bacteria and the host cell's nuclear DNA stained mainly with propidium. Scale bar = 7.5 µm. E. Specific PCR amplification of the *S. aureus* 16S rRNA gene (left panel) from cell lysates and the MLVF pattern (right panel) of bacteria infecting cells on consecutive days post-phagocytosis (numbers above lanes). Lane 0, sample collected immediately upon completion of phagocytosis (2h). Lane N, the MLVF pattern of *S. aureus* Newman before infection.

Cultured hMDMs engulfed *S. aureus* strain Newman efficiently, with the majority of bacteria being internalized by 2 h. To determine the intracellular survival/killing rate of *S. aureus* cells the infected hMDMs were lysed at specific time points post-phagocytosis and plated onto TSA. The intracellular survival of *S. aureus* was dependent on the MOI. At MOI = 5 or 10 all of the intracellular bacteria were killed by 3 days postphagocytosis (data not shown). Conversely, at MOI = 25 the number of viable intracellular staphylococci decreased gradually between day 1 and day 5 by approximately five orders of magnitude compared to day 0 (Fig. 3A). Significantly, the conditioned medium of the infected cultures was sterile throughout. This suggests that hMDM effectively killed the internalized *S. aureus*, and the surviving bacteria did not escape intracellular confinement. Nevertheless, at higher MOI macrophages could not completely eradicate the bacteria, since an explosion of bacterial growth occurred on day 6, with similarly large numbers of *S. aureus* free in the medium and apparently adherent to cell corpses. At this time all macrophages that had appeared healthy on the preceding day had lost their plasma membrane integrity. Significantly, the same kinetic of *S. aureus* intracellular survival was observed in hMDMs cultivated for both 1 and 2 weeks, eliminating the possibility of macrophage aging triggering bacterial release (data not shown).

A burst of live *S. aureus* in the conditioned media may indicate that the bacteria either suddenly proliferated intracellularly, with large numbers of bacterial cells being released into the media after hMDM lysis, or that a small number of surviving intracellular bacteria escaped intracellular confinement and multiplied vigorously in the media, killing the hMDMs in the process. To discern between these two vastly different possibilities we have simultaneously monitored live bacteria in media, and also cell membrane damage. In four independent experiments we found that proliferation of *S. aureus* in the conditioned medium clearly preceded any significant cell damage as measured by the release of LDH (Fig. 3B, left panel). This result was fully corroborated by staining cells with propidium, showing that plasma membrane permeabilization was secondary to the robust growth of *S. aureus* in the media (Fig. 3B, right panel). Collectively these data, together with kinetics of intracellular survival (Fig. 3A), argue that *S. aureus* persists inside hMDM in continuously decreasing numbers until the exhausted cells allow bacteria to escape into the conditioned medium, where they proliferate.

Experiments monitoring the survival of *S. aureus* strain Newman inside hMDMs were repeated a total of 76 times with cells obtained from a number of different donors. Despite some minor variations in CFU on individual days we found a consistently high level of phagocytosis, a reproducible decrease in cultivable bacteria 4–5 days post-phagocytosis, and the sudden growth of *S. aureus* on day 5–6 which occurred simultaneously with, or proceeding, macrophages lysis. An occasional deviation from the pattern shown in Fig. 3A was the proliferation of *S. aureus* on day 3 or 4 (11 cases). We also observed the complete elimination of bacteria (7 cases), and in a single case intracellular *S. aureus* had converted into small colony variant (SCV) that persisted in hMDMs without causing lysis (data not shown). These variations can be explained by differences amongst donors.

Analysis of the data in Fig. 3A indicates that only a small number of bacteria survived intracellular killing by macrophages. To confirm the presence of intracellular *S. aureus* within hMDMs at various times post-uptake, cells were fixed and stained with acridine orange (AO) and examined by confocal microscopy. As seen in Fig. 3C the number of intracellular bacteria decreased from day 1 to day 3, reaching the lowest level on day 5 which correlates with viable counts. We assessed whether intracellular bacteria at day 5 were viable by staining cells with the LIVE/DEAD BacLight Kit which differentiates live and dead *S. aureus* by staining the latter with a red fluorophore and viable bacteria with a green fluorophore (Fig. 3D). This showed that although the majority of bacteria were alive, a significant number of dead intracellular staphylococci were present. The presence of *S. aureus* within hMDMs was confirmed by the amplification of 16S rRNA PCR fragment, using *S. aureus-*specific primers, from total DNA isolated from the lysates of infected hMDMs (Fig. 3E, left panel). Finally, multi-locus VNTR fingerprinting (MLVF) [56] confirmed that hMDMs cultures were not contaminated with other strains of *S. aureus*. Identical banding patterns (Fig. 3E, right panel) indicated that strain Newman was present throughout.

### Priming macrophages with interferon-γ helps the cells eradicate intracellular *S. aureus*

**Figure 4:**
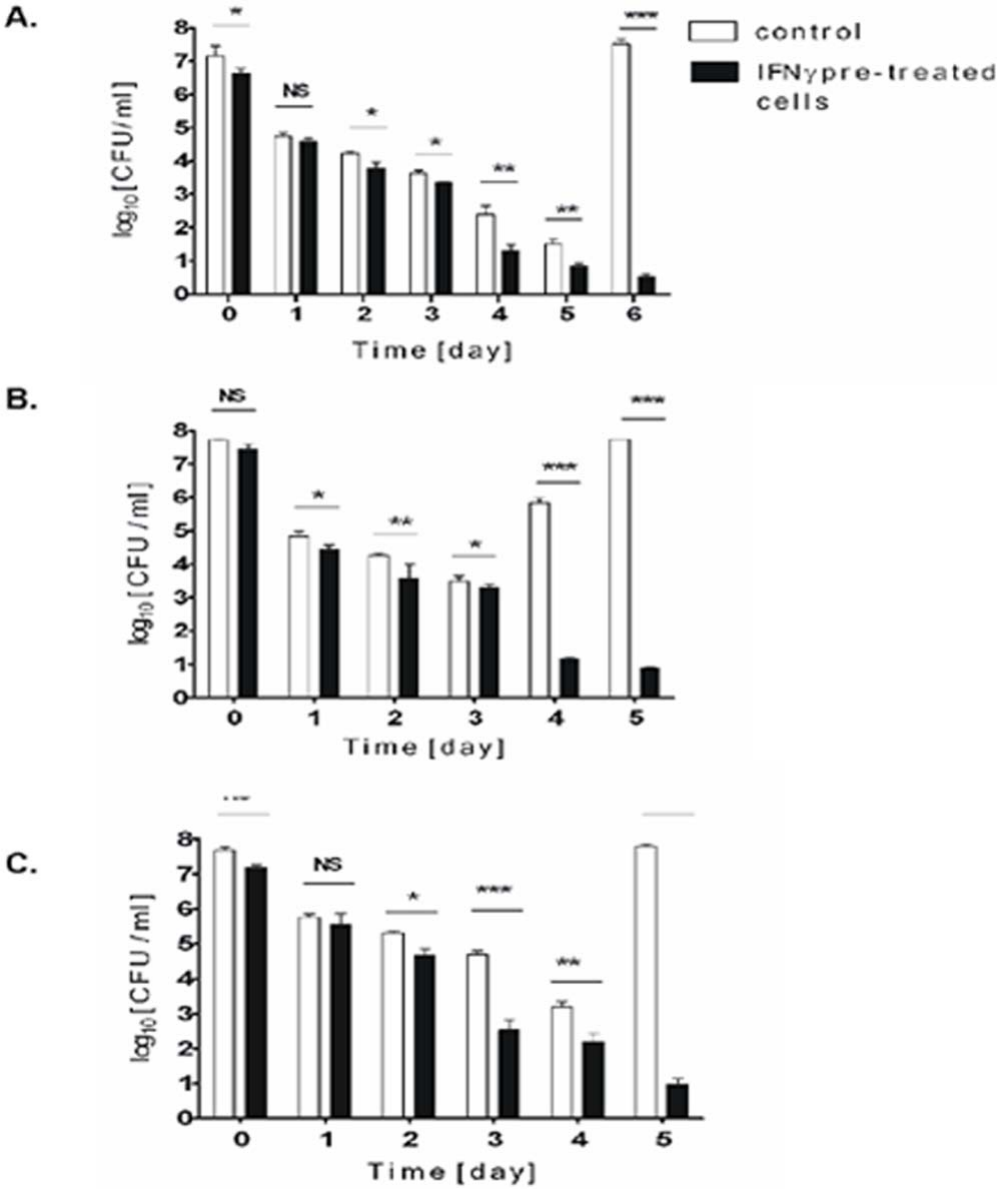
Macrophages stimulated with interferon-γ kill engulfed *S. aureus* strains: Newman (A), ATCC 25923 (B), and COL (C) more efficiently than non-stimulated cells. Cells were stimulated with recombinant human IFNγ overnight at concentrations equivalent to human therapeutic doses (100 U ml^−1^), and then allowed to phagocytose three different strains of *S. aureus* for 2 h. Infected cultures were processed as described in the legend for Fig. 3A and live bacteria in the whole cultures (CFU) were enumerated up to 14 days postphagocytosis. Since at the longer timepoints no bacterial growth was detected (CFU = 0) these points were not presented on the graph. The data shown is representative of at least three separate experiments, performed in triplicate, using hMDM derived from different donors. Bars represent mean CFU value ±SD. *, p<0.05; **, p<0.01; ***, p<0.001. NS-not significant.

Interferon-γ (IFNγ) plays an essential role in stimulating cells to eliminate intracellular pathogens [57]. Therefore we checked the effect of IFNγ on the course of hMDMs infection with three different *S. aureus* strains. As can be clearly seen in Fig. 4, from day 2 onwards there are significant differences between the CFU from the lysates of IFNγ-pretreated or non-treated macrophages. The most dramatic difference was observed on day 6 for Newman (Fig. 4A) and day 5 for strains ATCC 25923 and COL (Fig. 4BC). While *S. aureus* escaped from non-treated cells into the media, where proliferated explosively, IFNγ-stimulated cells curtailed the numbers of intracellular staphylococci, eliminating strain Newman on day 7, and strains ATCC 25923 or COL on day 6; yielding cultures that were sterile for up to 6 consecutive days (end of experiment) after the intracellular infection was cleared. These results demonstrate that as in the case of other intracellular pathogens [57], the infection of hMDMs by *S. aureus* can be resolved if macrophages are primed with IFNγ.

### *S. aureus* persists in intracellular vacuoles

**Figure 5:**
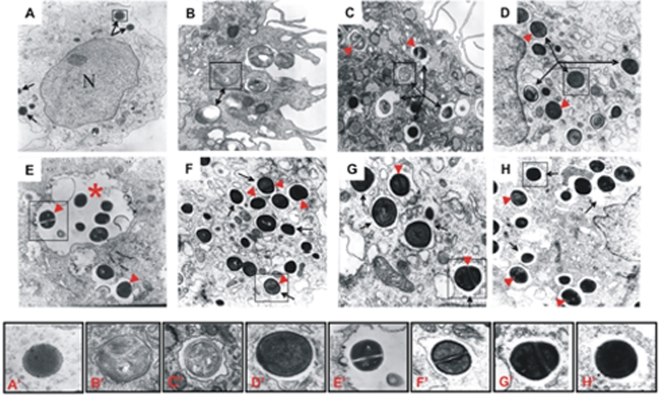
Apparently structurally normal, intact dividing and viable *S. aureus* Newman cells persist in the intracellular vacuolar compartment on four consecutive days post-phagocytosis. The photographs presented are representative of a minimum of 20 fields observed. hMDMs were allowed to engulf bacteria for 2h at a MOI of 25, and infected macrophages were fixed with Karnovsky's fixative either immediately following phagocytosis (2 h), or on consecutive days post-phagocytosis, before being processed by standard electron microscopic techniques. At any given point post-phagocytosis dividing bacterial cells were visible (arrowheads). Magnified views of selected bacteria framed on main micrographs are shown in the bottom row labeled from A' to H', respectively. A. Control, non-infected hMDM has a morphological appearance typical for that of professional phagocyte, with vesicles localized around the nucleus (N) and only few lipid droplets (arrows). Magnification: x14,000. B. Immediately post-phagocytosis (2h), the bacteria were located predominantly in very tight membranous compartments (arrows). Magnification: x50,000. C. Already 1 day post-phagocytosis the majority of *S. aureus* cells were found in clearly defined membrane-enclosed vacuoles (arrows). A partially degraded bacterium in the vacuole is framed. Magnification: x20,000. D and E. At day 2 the cells of *S. aureus* can only be found in well defined vacuoles (arrows), occasionally spacious vacuoles (asterisk) containing several bacteria. Magnification: x32,500. F. On day 3 *S. aureus* persists in vacuoles which often reveal signs of partial membrane discontinuity (arrows). Magnification: x32,500. G and H. On day 4, the majority of bacteria were found in vacuoles with profoundly damaged or fully disintegrated membranes (arrow). Magnification: x54,000 (G) and x32,000 (H).

The strategies employed by obligate intracellular pathogens to avoid intracellular killing by professional phagocytes can be broadly categorized as immediate escape from the phagosome into the cytoplasm or modification of the phagosome preventing it from fusing with lysosomes [58], [59]. In order to investigate the intracellular localization of staphylococci we have analyzed hMDMs infected with *S. aureus* strain Newman by transmission electron microscopy. Macrophages engulfed the bacteria by forming phagocytic cups (Fig. 5B). At 2h post-inoculation (Fig. 5B) internalized *S. aureus* cells could be seen in very tight membranous compartments (71.2% of internalized bacteria), or in clearly defined vacuoles (28.8%). After only 24 h the majority of *S. aureus* cells (61.2%) were located in single, membrane-bound vacuoles (Fig. 5C). At 2–3 days post-phagocytosis staphylococci were found exclusively inside vacuoles (Fig. 5DEF) some of which were spacious and occupied by several bacterial cells (Fig. 5E). Significantly, on day 4 considerable numbers of bacteria were found in vacuoles whose membranes were partially or totally degraded (Fig. 5GH). Finally, it is important to underline that at any point post-infection, dividing intact bacteria were frequently observed in vacuoles. Collectively these results strongly support our contention that *S. aureus* can survive intracellular killing inside vesicular compartments.

### Intracellular survival of *S. aureus* depends on functional *agr* and *sigB* loci, but not on *sarA*

**Figure 6:**
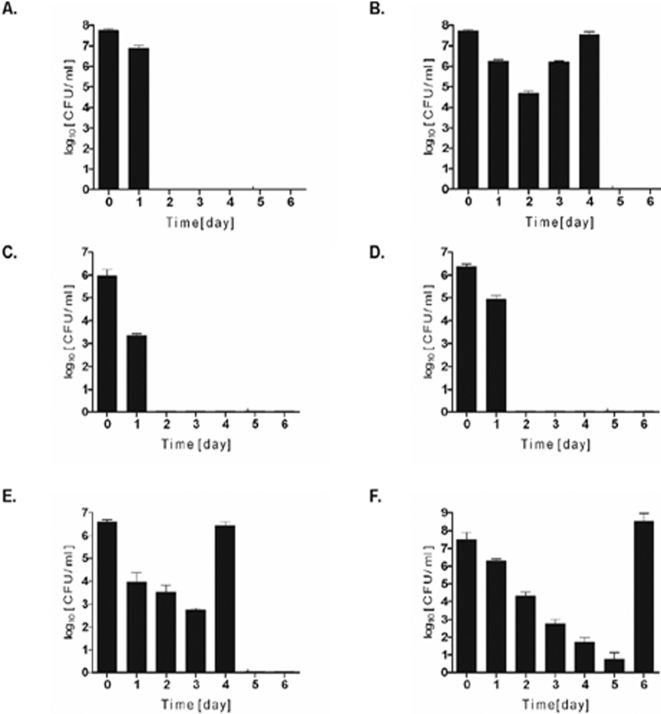
Functional *sigB* and *agr* operons, but not *sarA*, are indispensable for *S. aureus* to survive phagocytosis by hMDMs. Macrophages were allowed to engulf defined strains of *S. aureus* for 2h at a MOI of 25, and the intracellular survival of bacteria on consecutive days post-phagocytosis was monitored by enumeration of the CFU of cell lysates (see Fig. 3A legend). The data shown is representative of at least three separate experiments, performed in triplicate, using hMDMs derived from different donors. Bars represent mean CFU value ±SD. A. 8325-4, a natural *rsbU* defective strain lacking a functional SigB. B. SH1000, a derivative of strain 8325-4 with a restored *rsbU* gene and SigB activity. C. Newman *sigB* mutant D. Newman *agr* mutant E. Newman *sarA* mutant F. Newman wild-type

The intracellular survival of *S. aureus* in neutrophils, as well as in non-professional phagocytes, depends on multiple virulence factors, including global regulators of gene expression [32], [40]. Therefore we compared the uptake and intracellular killing/survival of *S. aureus* strains deficient in *agr*, *sarA* or *sigB*. First we examined the survival of *S. aureus* strain 8325-4 in which an 11bp deletion in the *rsbU* gene renders the *sigB* operon essentially non-functional [60]. Macrophages cleared intracellular 8325-4 within two days, apparently by killing the bacteria in phagolysosome (Fig. 6A). The importance of *sigB* was confirmed by the finding that *S. aureus* strain SH1000, 8325-4 with a restored *rsbU* gene and a functional *sigB* operon [61], survived phagocytosis (Fig. 6B). Furthermore, a Newman *sigB* mutant was unable to survive phagocytosis, with all ingested bacteria killed within 2 days (Fig. 6C).

The significance of *agr* and *sarA* was studied using isogenic mutants of *S. aureus* Newman. The inactivation of *agr* eliminated the ability of *S. aureus* to survive within hMDMs. By the second day of infection no viable bacteria were apparent in cell lysates, indicating that the engulfed *S.* *aureus* had been completely destroyed (Fig. 6D). Somewhat surprisingly, inactivation of *sarA* had no effect on intracellular survival (Fig. 6E) and the *sarA* mutant had a pronounced tendency to lyse the cells two days earlier than the parental strain (Fig. 6F). Taken together these results argue that overlapping, but seemingly distinct, subsets of genes are crucial for *S. aureus* persistence in hMDMs and other cell types, including neutrophils and non-professional phagocytes.

### Expression of α-toxin, MSCRAMMs, and aureolysin, but not other proteases, contributes to *S. aureus* persistence in macrophages

**Figure 7:**
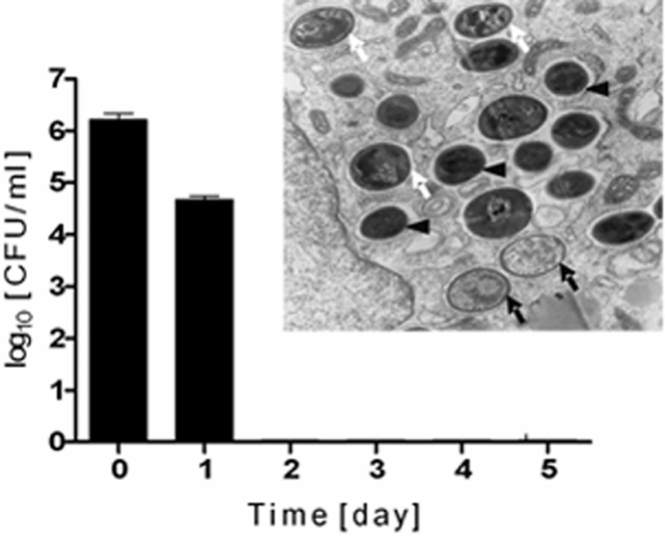
An α-hemolysin mutant of *S. aureus* strain Newman is efficiently killed in phago(lyso)somes within two days of phagocytosis. Intracellular persistence of *S. aureus* was determined as described in the legend of Fig. 3A. Bars represent mean CFU values ±SD and are representative of at least three separate experiments, performed in triplicate, using hMDMs derived from different donors. An electron microphotograph of infected hMDMs was obtained 1 day post-phagocytosis and shows partially (white arrows) and totally degraded (black arrows) bacterial cells inside tight vacuoles. At this time point some apparently intact bacterial cells are also present (black arrowheads). Magnification: x32,500. The photograph presented is a representative of a minimum of 20 fields observed.

**Figure 8:**
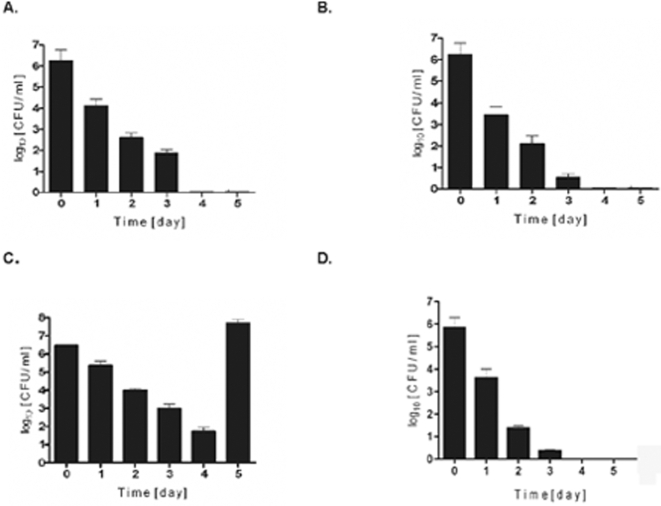
Metalloprotease (Aur), protein A, and MSCRAMs, but not the V8 protease (SspA) and staphopain B (SspB), contribute to *S. aureus* intracellular persistence in infected hMDMs. Intracellular persistence of *S. aureus* was determined as described in the legend of Fig. 3A. Bars represent mean CFU value ±SD and are representative of at least three separate experiments, performed in triplicate, using hMDMs derived from different donors. A. Newman *aur* mutant B. Newman *srtA* mutant C. Newman *ssp* operon mutant D. Newman *spA* mutant

SarA and *agr* differentially regulate the expression of virulence factors, especially surface-associated proteins (MSCRAMMs) and secreted enzymes [8]. In addition, it has previously been established that α-hemolysin is vital for the escape of *S. aureus* from phagosomes into the cytoplasm of infected, non-professional phagocytes [15], [62]. Therefore we examined the effects of inactivation of *hla* (Fig. 7), the major extracellular proteases (aureolysin, the V8 protease and staphopain B), and surface adhesins (via a sortase A mutant) (Fig. 8) on the survival of *S. aureus* Newman phagocytosed by hMDMs.

In comparison to the wild-type, which persisted intracellularly for 5 days before lysing macrophages, *hla*-deficient mutant was eradicated by day 2 (Fig. 7). TEM examination of macrophages directly after phagocytosis did not show any significant difference in the intracellular distribution of wild-type or *hla* mutant cells (data not shown). Significantly, however, in contrast to the parental strain, on day 1 the majority of *hla* mutant cells were found to be either partly or completely degraded (Fig. 7, inset) which correlates well with the lack of viable *S. aureus* at day 2.

Of the three major extracellular proteases only aureolysin was shown to have a role in the survival of *S. aureus* inside macrophages (Fig. 8A). The *ssp* mutant, deficient in both the V8 protease (SspA) and staphopain B (SspB), had a phenotype that was indistinguishable from the parental strain (Fig. 8C). In comparison to *agr*, *sigB* and *hla* mutants (see Figs. 6 and 7), the rate of intracellular killing of the *aur* mutant was slower, with live staphylococci consistently present in cell lysates up to day 3. Also, macrophages infected with a sortase A mutant, which is deficient in multiple MSCRAMMs, cleared bacteria more slowly (Fig. 8B) at a rate that was comparable to that of the *aur* mutant. Interestingly, the *S. aureus* protein A deficient mutant , was killed by macrophages with kinetics remarkably similar to those of the sortase A mutant (compare Fig. 8A and 8D). Significantly, the lack of MSCRAMMs did not affect the internalization of *S. aureus* cells since there were no statistically significant differences between the number of initially phagocytosed MSCRAMM-deficient cells versus the number of wild-type or the *aur* or *ssp* mutants.

## Discussion

In this study we detail findings that confirm previous observations that *S. aureus* can survive phagocytosis by macrophages [45], [47], [49]. Most importantly, however, our data shows for the first time that this bacterium can not only persist for several days inside macrophages, but ultimately is able to escape the intracellular confinement and proliferate rapidly in the conditioned media. Throughout the course of intracellular infection with each *S. aureus* strain tested (Newman, COL, and SH1000) macrophages showed no signs of necrosis until the day of lysis. In the case of strain Newman, the plasma membrane of infected macrophages maintained their integrity for up to 5 days with mitochondria remaining functional and cells actively metabolizing lipids (Fig. 1). Over the course of the infection macrophages appeared to be able to reduce the initial intracellular bacterial load by several orders of magnitude, in conjunction with a maintained ability to produce reactive oxygen species. Eventually, however, macrophages succumbed to the infection and were killed. In stark contrast to epithelial, endothelial and mesothelial cells, enterocytes, keratinocytes, osteoblasts, lymphocytes [17], [63]–[65] and especially human monocytes [66], [67], the macrophages infected by *S. aureus* did not undergo apoptosis. At numerous time points several tests were applied to detect apoptosis, all of which were negative, despite initial transient phosphatidyl serine exposure on the cell surface (annexin V binding) and procaspase 3 activation (Koziel *et al*.–manuscript in preparation). Taken together these data suggest that by escaping phagolysosomal killing, *S. aureus* may be able to manipulate macrophages differentially compared to the other cell types in which it triggers apoptosis. However, it must be kept in mind that although hMDMs are an excellent model for *in vitro* studies, they are not tissue or inflammatory macrophages, and consequently the *in vivo* interaction of *S. aureus* with these professional phagocytes is affected by a multitude of pathophysiological factors.

The kinetics of intracellular killing of bacteria, and the timing of the escape of internalized *S.* *aureus* into the conditioned medium were consistent and reproducible for a given staphylococcal strain, despite the fact that macrophages were derived from many different donors. The occasional deviations from typical patterns of intracellular survival and cell lysis (Fig. 3A) were likely the result of donor variation.

By examining TEM micrographs of infected macrophages several important observations were made. First, *S. aureus* was engulfed by the formation of typical phagocytic cups (Fig. 5B). This efficient phagocytosis was apparently carried out by a class A and B (CD36) scavenger receptors with the assistance of serum proteins present in the culture media. These proteins act as opsonins by binding to the bacterial cell surface, facilitating interaction with specific phagocyte receptors, as well as promoting bacterial engulfment [68]–[70]. Second, immediately after completion of phagocytosis the majority of *S. aureus* cells occurred in tight vacuoles, some of which were apparently later converted into spacious membranous compartments. Akin to this, two types of phagocytic vacuoles were also noticed in a study on the phagocytosis of *S. aureus* by neutrophils, where it was suggested that effective bacterial killing only occurred within tight phagosomes [40]. The same would appear to be true of macrophages, and is consistent with the observation that the rapidly-eradicated *S. aureus hla* mutant was only found in tighter vacuoles (Figs. 5 and 7). Third, at all stages seemingly intact *S. aureus* cells that were often in the process of dividing persisted in vacuolar compartments. Finally, as the time of macrophage lysis approached we observed an increasing number of bacteria-bearing vacuoles whose membranes were at differing stages of degradation. TEM findings as well as data obtained from the confocal microscopy imaging showed many living bacterial cells in the macrophages (Fig. 3D).

In comparison to well studied intracellular pathogens such as *Shigella flexneri*, *Chlamydia* spp., *Mycobacterium* spp., *Legionella pneumophila*, *Listeria monocytogenes*, *Salmonella* spp., and *Brucella abortus* we know little about how *S. aureus* can survive phagocytosis. Recently, it has been shown that the subversion of autophagy by *S. aureus* internalized by HeLa cells and fibroblasts can be central to intracellular endurance in non-professional phagocytes [27]. However, in the case of macrophages, we have neither observed in our TEM micrographs multilamellar structures attached to, or fused with the membranes of *S. aureus* vacuoles, nor double membranes enveloping the bacteria which are the main morphological criteria for autophagosomes. Conversely, *S. aureus* was consistently found inside well defined single-membrane vacuoles.

Intracellular persistence of *S. aureus* in non-professional phagocytes depends on the expression of the global regulatory locus *agr*, and on the alternative sigma factor, *sigB*, but not on *sarA* [32]. In contrast to our findings with macrophages it has previously been demonstrated that *sarA* is required for the survival of staphylococci upon phagocytosis by neutrophils [40]. SarA was not only dispensable for intracellular persistence in human macrophages, but the *sarA* mutant survived inside phagocytes with higher numbers of viable cells and initiated proliferation at least one day prior to that of the parental strain (Fig. 6). This effect may well be attributable to the pleiotropic regulatory activity of SarA.

In contrast to the *sarA* mutant, strains lacking SigB were efficiently killed with bacteria being totally eradicated within 48 h. The importance of SigB was emphasized when comparing 8325-4, which contains a natural mutation in the *rsbU* locus that renders *sigB* essentially non-functional, and its derivative SH1000, which has restored SigB function. Strain 8325-4 was unable to survive engulfment by macrophages whereas SH1000 was the most aggressive of all *S. aureus* strains tested in surviving this challenge. It is not yet possible to identify which factors controlled by SigB are vital for *S. aureus* survival since it is a pleiotropic regulator that modulates the expression of large numbers of genes, including those encoding virulence factors and proteins involved in the environmental stress response [71], [72]. Strains defective in *sigB* lack the golden pigmentation that is characteristic of *S. aureus*, which has been shown to be essential in protecting *S. aureus* against reactive oxygen species [73]. It is possible that lack of pigment may be directly responsible for the efficient killing of strain 8325-4 and the Newman *sigB* mutant.

The *agr* locus has previously been shown to be absolutely essential for the intracellular survival of *S. aureus* in a variety of different cell types by facilitating the escape of bacteria from the endosome into the cytoplasm [32], [33], [74]. Here we show that it is indispensable for survival in macrophages. Although at least 100 genes are differentially regulated by *agr* [75], it's most significant impact on intracellular survival may be via the regulated expression of α-toxin. However, while *S. aureus*, with the help of α-toxin, escapes endosomes within hours of engulfment by non-professional phagocytes, it persists in membranous compartments in macrophages for up to 5 days. This suggests that *hla* expression by *S. aureus* inside macrophages may be regulated differentially compared to other cell types where significant amounts of toxin are produced immediately following invasion facilitating the prompt escape of bacteria into the cytoplasm and eventually triggering cell death by activation of caspase-dependent or independent apoptosis [30], [76]–[78]. α-toxin may work at two different stages by first facilitating disintegration of the vacuolar membranes that confine *S. aureus* and second by lysing the plasma membrane and facilitating escape from the cytoplasm into the conditioned medium. The effective eradication of the *S. aureus hla* mutant before any visible damage to the phagosomal membrane argues that lysing both vacuolar and cytoplasmic membranes is a major role of this toxin.

The metalloprotease aureolysin, the V8 serine protease (SspA) and staphopain B (SspB), are each produced *in vivo* following phagocytosis by human neutrophils [77] but only the first contributed to the survival of *S. aureus* in macrophages. Nevertheless, it cannot be excluded that SspA and SspB have an effect on *S. aureus* survival because they are activated in a cascade-like manner, initiated by aureolysin [79]. In the absence of the metalloprotease, SspA and SspB occur as proteolytically-inactive zymogens. Possibly the proteases retard endosome maturation by cleaving one or more host protein. Alternatively, they may target proteins regulating the antibacterial response of macrophages. Both these possibilities are the focus of ongoing research in our laboratories.

Finally, the anchoring of MSCRAMMs to the cell wall also contributes to *S. aureus* survival and/or escape from intracellular confinement as the sortase A-deficient mutant of strain Newman was efficiently killed by macrophages. The observation that the *S. aureus* protein A deficient strain was attenuated in intracellular persistence to the same degree as the *srtA* mutant, suggests the importance of protein A in surviving confrontations with macrophages. This is in agreement with the known importance of protein A for *S. aureus* virulence [80]. Additional surface protein(s) may also contribute to survival, but the fact that Newman is defective in its net fibronectin binding capacity due to point mutations in the two major fibronectin-binding genes (*fnbA* and *fnbB*) [81], argues against the importance of these adhesins in the process of intracellular persistence of *S. aureus* in macrophages.

In summary, it is evident that the mechanism for survival of *S. aureus* inside macrophages differs considerably from its interactions with other cells types. *S. aureus* preserves the integrity of the macrophage, using it as an intracellular niche, rather than destroying it. Nothing is yet known about how macrophages affect transcriptional regulation of intracellular *S. aureus* or why bacteria do not quickly escape from phagosomes as they do from other cells. However from the data presented here, it is clear that intracellular *S. aureus* can survive within human macrophages for several days until a point where bacteria escaped the intracellular confinement, proliferated in the conditioned medium and killed cells. In comparison to the more stealthy strategy of conversion to small colony variants, which allows for survival for months or even years [82], it would appear that hiding within macrophages is a short-term solution. Nevertheless, it may provide *S. aureus* with an advantage since, as is the case for *L. monocytogenes* and *M. tuberculosis*, infected mobile macrophages can serve as vehicles for the dissemination of infection [83]–[85]. This would explain the ability of *S. aureus* to invade the circulation from localized sites of infection and to disseminate systemically.

## Materials and Methods

### Reagents

Acridine orange, propidium iodide, ethidium bromide, Bodipy 493/503 were from Molecular Probes (Invitrogen). FCS (Fetal Calf Serum), gentamycin, erythromycin, tetracycline, chloramphenicol, sodium cacodylate trihydrate, RNase A, proteinase K, bovine serum albumin (BSA), saponin, recombinant human interferon γ (IFNγ), tryptic soy agar (TSA), tryptic soy broth (TSB), lysostaphin and agarose were obtained from Sigma. RPMI 1640 and CMF-PBS (without Ca^2+^ and Mg^2+^) and Ficoll-Paque were obtained from Gibco and Amersham Bioscience, respectively. LDH Kit was obtained from Promega. Genomic DNA Prep Plus Kit was purchased from A&A Biotechnology and Taq recombinant polymerase was obtained from MBI Fermentas.

### Cell culture

hMDMs were obtained from peripheral blood mononuclear cells (PBMCs). Briefly, PBMCs were isolated from human blood using a Ficoll-Paque density gradient and plated at 3×10^6^/well in 24-well plates (Sarstedt) in RPMI1640 supplemented with 2 mM L-glutamine, 50 µg ml^−1^ gentamycin (Sigma), and 10% autological human serum. After 24 h, non-adherent PBMCs were removed by washing with complete medium, and adherent cells were routinely cultured in this medium for 7 days with fresh medium changed every 2 days [86]. For some experiments hMDMs were cultured for 14 days.

### Bacterial strains, storage and growth conditions

The laboratory *S. aureus* strains used in this study are listed in Table 1. The strains were stored in TSB medium containing glycerol (50% v/v) at −80°C. Cultures were inoculated from stocks into 10 ml media. *S. aureus* strains were grown overnight under constant rotation (180 rpm) to stationary growth phase at 37°C. If required, media were supplemented with antibiotics at the following concentration: erythromycin at 5 µg ml^−1^ or 10 µg ml^−1^; tetracycline at 2 µg ml^−1^. The bacterial cells were collected by centrifugation (5,000×g, 8 min), washed with phosphate-buffered saline (PBS) and resuspended in PBS to the desired OD_600nm_. For some experiments, staphylococci were opsonized by incubation at 37°C for 30 min in heat inactivated FCS, washed and resuspended in PBS. Accuracy of preparation of bacterial samples for phagocytosis assay was routinely verified by plating dilutions on agar plates and counting colonies to determine CFU per ml. Occasionally, viability of staphylococci used to incubate with hMDMs was evaluated using a LIVE/DEAD® BacLight™ bacterial viability kit from Molecular Probes and found >90% viable.

**Table 1:** Bacterial strains.

*S. aureus* strains	Relevant genotype/markers	Relevant properties	Source/reference
ATCC 25923		clinical isolate	ATCC
Newman		wild type laboratory strain	T.J. Foster
COL		wild type laboratory strain	S. Foster
8325-4		NCTC 8325 cured of prophages	S. Foster
SH1000		*rsbU* ^+^ 8325-4 derivative	S. Foster
Newman (*sarA^−^*)	*sarA*::Em^R^	*sarA* ^−^; isogenic mutant of Newman	T.J. Foster
Newman (*agr^−^*)	*agr*::Tc^R^	*agr^−^;* isogenic mutant of Newman	T.J. Foster
Newman (*sigB^−^*)	*sigB*::Tc^R^	*sigB* ^−^; isogenic mutant of Newman	T.J. Foster
Newman (*hla^−^*)	*hla*::Em^R^	*hla* ^−^; isogenic mutant of Newman	T.J. Foster
Newman (*aur^−^*)	*aur*::Em^R^	*aur* ^−^; isogenic mutant of Newman	T.J. Foster
Newman (*sspA^−^*)	*sspA*::Em^R^	*sspA* ^−^; isogenic mutant of Newman	T.J. Foster
Newman (*srtA* ^−)^	*srtA*::Em^R^	*srtA* ^−^; isogenic mutant of Newman	O. Schneewind
Newman (*spA* ^−)^	unmarked	*spA* gene deleted	T.J. Foster

### Phagocytosis assay and samples preparations

A suspension of *S. aureus* in PBS was added to 3×10^5^ hMDMs cultured in RPMI1640 medium containing L-glutamine and 10% heat inactivated human autologous serum in the 24-well culture plate to the final amount of 1.5×10^6^ CFU, 3×10^6^ CFU, 7.5×10^6^ CFU, 1.5×10^7^ CFU, and 3×10^7^ CFU (the ratio bacteria : hMDM = from 5∶1 to 100∶1, multiplicity of infection, MOI = 5, 10, 25, 50 and 10, respectively). hMDMs and staphylococci were co-cultured for 2h in a humidified atmosphere containing 5% CO_2_. Phagocytosis was stopped by putting the plates on ice and washing the hMDMs cultures twice with ice-cold PBS to remove non-phagocytosed bacteria. Any remaining extracellular bacteria were killed by overnight culturing hMDMs in RPMI1640 medium containing gentamycin (50 µg ml^−1^). Then, the medium was changed again to fresh media without antibiotic and the cultures were maintained for up to 8 days with medium changes every second day.

Initial samples for determination of colony-forming ability of *S. aureus* present in culture medium and internalized by macrophages, as well as samples for microscopic examinations, PCR analysis, and hMDMs viability assessment were all taken immediately after cells were washed out of free non-phagocytosed bacteria, before medium was supplemented with gentamycin. Consecutive samples were taken every 24h for up to 8 days or until *S. aureus* escaped into the conditioned medium and cells were lysed as evaluated by light microscopical inspection. All samples were prepared in triplicates.

Since the opsonization of *S. aureus* with heat-inactivated serum (inactive complement) prior to bacterial co-culturing with macrophages did not affect the rate of phagocytosis (data not show) we assumed that the presence of serum in the culture medium during phagocytosis yielded enough opsonins to facilitate the efficient phagocytic uptake of *S. aureus* by macrophages. Therefore all experiments described in this paper were performed without prior opsonization.

### IFN γ treatment

hMDMs were preincubated overnight with 100 U/ml interferon γ (activated hMDMs). Then, the cells were exposed to *S. aureus* (Newman, ATCC 25923 or COL strain) at MOI 25. Simultaneously, the non-stimulated cells were incubated with the same *S. aureus* strain and regarded as a control. After 2 hours of phagocytosis, extracellular bacteria were killed by adding the gentamycin to the culture medium and the cultures were continued as described above. Directly after phagocytosis and at consecutive days post-phagocytosis, intracellular bacteria released by cell lysis were analyzed for colony forming ability.

### Assay for colony-forming ability of engulfed bacteria

PBS-washed hMDMs directly after 2h incubation with *S. aureus* and infected cell cultures at consecutive days post-phagocytosis were lysed by treatment with ice-cold water. The lysates were plated at serial dilutions in sterile PBS on TSB agar plates. The plates were incubated at 37°C overnight or for 2 days and the number of colonies (CFU) was enumerated. In parallel, the presence of viable staphylococci in the conditioned media was also determined by the colony-forming assay.

### Lactate Dehydrogenase (LDH) measurement-Cytotoxicity Assays

To assess the effect of *S. aureus* on cell necrosis, the LDH release assay was performed using the CytoTox96 nonradioactive cytotoxicity assay kit (Promega) according to the manufacturer's instructions. Cytotoxicity was calculated with the formula: % cytotoxicity = (experimental value−low control)×100/(high control–low control), where low control is assay medium plus cells and high control is assay medium (plus 2% Triton X-100) plus cells to define the maximum LDH release. The spontaneous release was always up to 10% of the maximum release. Relative amounts of LDH release was measured (absorbance at 490 nm) using an ELISA plate reader. All assays were performed in triplicate.

### MTT Cell Proliferation and Cytotoxicity Assay

To quantify mitochondrial activity of infected hMDMs the MTT tetrazolium dye reduction assay was applied. Cultures of control and *S. aureus* infected hMDMs at different times post-phagocytosis were incubated with the MTT (monotetrazolium) reagent (Sigma) for 3 h at 37°C. Then, a detergent solution was added to lyse the cells and solubilize crystals of formazan produced by mitochondria. The samples were read using an ELISA plate reader at a wavelength of 570 nm. The amount of color produced was directly proportional to the number of viable cells. All assays were performed in triplicate.

### Respiratory burst assay

Control hMDMs, and *S. aureus*-infected cells on 4^th^ day post-phagocytosis, were washed twice with PBS and scraped into TBS buffer. Cell suspensions (0.5×10^6^ in 0.6 ml of TBS) were incubated at 37°C for 5 min with 10 µM 2′,7′-dichlorodihydrofluorescein diacetate (DCFH-DA) obtained from Molecular Probes, Inc. (Leiden, The Netherlands). Next, live *S. aureus* (10^7 ^CFU) or latex beads (1.7–2.2 µm, Sphero Nile Red BD Bisciences Pharmingen) were added to macrophage suspensions. At differing time intervals aliquots were withdrawn and subjected to flow cytometric analysis. The fluorescence and light-scattering properties of cells was determined using FACScan Cytometer (Becton Dickinson). The green fluorescence of DCF was observed using wavelengths of 521 nm (emission), and 488 nm (excitation). The mean fluorescence intensity of cells was quantitatively analyzed.

### Fluorescence staining and confocal microscopy

hMDMs cultured on glass coverslips were placed into 6 well plates. Both, control and infected with *S. aureus* (*Newman* strain) cells were fixed for 30 min at room temperature with 8% paraformaldehyde in 200 mM HEPES, pH 7.4, then washed with CMF-PBS. The fixed cells were treated with RNase A at 4°C, overnight. After incubation with RNase, 10 µg ml^−1 ^acridine orange (AO) was added to the samples. Green fluorescence corresponds to nucleic acids of host cell DNA as well as to nucleic acids of bacteria. Cells were examined using the Bio-Rad MRC1024 confocal system, interfaced with a Nikon Diaphot (Nikon, Amsterdam) inverted microscope equipped with argon and krypton-argon lasers, 3 detection channels and a Nikon PlanApo 60x oil immersion lens. Images were taken in the line-averaging mode and at a pinhole setting of one Airy unit. Every fluorescence image shown is an extended focus calculated as the zero-projection from stacks of single images taken at an interval of 0.2 µm. Image resolution was 512×512 pixels. AO interacts with DNA and RNA, having green fluorescence with an emission maximum at 525 nm when bound to DNA; upon association with RNA, its emission is shifted to ∼650 nm (red luminescence). Also, the cells were stained with propidium iodide (PI) at a final concentration 5 µg ml^−1^. Propidium binds to DNA and RNA, having red fluorescence with an emission maximum at 620 nm. The cells were also stained with BODIPY493/503 at final concentration 5 µM. The BODIPY fluorophore is specific for neutral, cytoplasmic lipids and used as tracer for lipid droplets and nonpolar liquids. Images were recorded using a LSM 510 Meta confocal laser scanning microscope (Carl Zeiss GmbH, Oberkochen, Germany) equipped with helium-neon and argon lasers. To distinguish vivid bacteria with intact cell walls from dead bacteria with compromised membranes, the LIVE/DEAD *Bac*Light Bacterial Viability kit was used. The kit provides two different nucleic acid stains-SYTO 9 and propidium iodide, which, mixed in proper ratios, give fluorescence signals indicative of alive or dead bacteria. Cells were permeabilized with 0.2% Triton X-100 to allow PI to bind to the dead bacteria and macrophage DNA (Fig. 3D). Green excitation (514 nm) light was used for SYTO 9 and PI staining.

### Transmission electron microscopy

Infected or non-infected adherent hMDMs cultured on 6-well plates, were trypsinized with 10% trypsin-EDTA (Sigma). After 10 minutes at 37°C, the cells were washed with ice-cold PBS and pelleted at 4°C. The cells were then fixed with Karnovsky solution (2% paraformaldehyde, 2.5% glutaraldehyde in 0.2 M sodium cacodylate buffer, pH 7.4, 30 min at room temperature, washed in the same buffer lacking the fixative and post-fixed in 2% osmium tetraoxide for 1h [87]. The pellets were dehydrated through serial incubation in graded series of ethanol solutions (30%, 60%, 90%, and 100%) and finally embedded in Epon. The sections were counter-stained with uranyl acetate, lead citrate and examined with a Zeiss electron microscope.

### MLVF (Multiple-locus VNTR fingerprinting) and PCR analysis

Total DNA of the isolates for PCR and MLVF typing were performed as described by Sabat *et al.* [56]. For DNA extraction, cell lysates were resuspended in 100 µl of lysis buffer (6 mM Tris-HCl, pH 7.6, 1 M NaCl, 0.1 M Na_2_EDTA, 0.5% Brij 58, 1% N-lauroylsarcosine sodium salt). Lysostaphin and RNase A were then added to concentrations of 50 µg ml^−1^ and 60 µg ml^−1^, respectively, and the mixture was incubated at 37°C for 30 minutes. A Genomic DNA Prep Plus kit used according to the manufacturer's instructions was applied to purify total DNA. The purified DNA was diluted 1∶10 with sterile water and stored at −20°C until required. PCR primers are listed in Table 2. The primers were designed based on the completed and unfinished *S. aureus* genomes available from The Institute for Genomic Research (http://www.tigr.org) (COL strain), the University of Oklahoma (http://www.genome.ou.edu) (strain 8325), the Sanger Institute (http://www.sanger.ac.uk) (strains MRSA252 and MSSA476), and Juntendo University (http://www.juntendo.ac.jp) (strains Mu50 and N315). Multiplex amplifications were performed in mixtures containing 1.5 mM MgCl_2_, 0.2 mM dNTPs and 0.05 U of *Taq* recombinant polymerase per µl. The final reaction volume of 20 µl contained 0.5 µM each of clfB-F and clfB-R, 1 µM each of clfA-F, clfA-R, sdrCDE-F, sdrCDE-R, spa-F, spa-R, sspA-F and sspA-R as well as 1 µl template DNA. The amplification of DNA fragments was performed with an initial denaturation (94°C, 5 min), followed by 20 cycles of denaturation (94°C, 30 sec), annealing (55°C, 30 sec) and extension (72°C, 30 sec) with a single final extension step (72°C, 5 min). The amplified products were separated using a 2% TAE agarose gel (Sigma) and visualized by staining with ethidium bromide. The amplification of 16S rRNA fragments was run: a denaturation step at 94°C for 3 minutes, followed by 30 cycles of 30 sec at 94°C, 60 sec at 45°C, 10 sec at 72°C, with a final extension at 72°C for 3 minutes.

**Table 2:** Oligonucleotides used in this study.

Oligonucleotide	Sequence
clfA-F	5′-GATTCTGACCCAGGTTCAGA
clfA-R	5′-CTGTATCTGGTAATGGTTCTTT
clfB-F	5′-ATGGTGATTCAGCAGTAAATCC
clfB-R	5′-CATTATTTGGTGGTGTAACTCTT
sdr-F	5′-GTAACAATTACGGATCATGATG
sdr-R	5′-TACCTGTTTCTGGTAATGCTTT
spa-F	5′-AGCACCAAAAGAGGAAGACAA
spa-R	5′-GTTTAACGACATGTACTCCGT
ssp-F	5′-ATCMATTTYGCMAAYGATGACCA
ssp-R	5′-TTGTCTGAATTATTGTTATCGCC
16S rRNA-F	5′-GAAAACTTGAGTGCAGAAGAGGAAAGTGG
16S rRNA-R	5′-GATGTCAAGATTTGGTAAGGTTCTTCGCG
